# Impact on house staff evaluation scores when changing from a Dreyfus- to a Milestone-based evaluation model: one internal medicine residency program's findings

**DOI:** 10.3402/meo.v19.25185

**Published:** 2014-11-24

**Authors:** Karen A. Friedman, Sandy Balwan, Frank Cacace, Kyle Katona, Suzanne Sunday, Saima Chaudhry

**Affiliations:** 1 Department of Internal Medicine, Hofstra North Shore LIJ School of Medicine, Hempstead, New York, NY, USA; 2 Department of Psychiatry, Hofstra North Shore LIJ School of Medicine, Hempstead, New York, NY, USA

**Keywords:** Medical Education-Graduate, Medical Education-assessment methods, Milestones, Next Accreditation System, ACGME core competencies

## Abstract

**Purpose:**

As graduate medical education (GME) moves into the Next Accreditation System (NAS), programs must take a critical look at their current models of evaluation and assess how well they align with reporting outcomes. Our objective was to assess the impact on house staff evaluation scores when transitioning from a Dreyfus-based model of evaluation to a Milestone-based model of evaluation. Milestones are a key component of the NAS.

**Method:**

We analyzed all end of rotation evaluations of house staff completed by faculty for academic years 2010–2011 (pre-Dreyfus model) and 2011–2012 (post-Milestone model) in one large university-based internal medicine residency training program. Main measures included change in PGY-level average score; slope, range, and separation of average scores across all six Accreditation Council for Graduate Medical Education (ACGME) competencies.

**Results:**

Transitioning from a Dreyfus-based model to a Milestone-based model resulted in a larger separation in the scores between our three post-graduate year classes, a steeper progression of scores in the PGY-1 class, a wider use of the 5-point scale on our global end of rotation evaluation form, and a downward shift in the PGY-1 scores and an upward shift in the PGY-3 scores.

**Conclusions:**

For faculty trained in both models of assessment, the Milestone-based model had greater discriminatory ability as evidenced by the larger separation in the scores for all the classes, in particular the PGY-1 class.

Graduate medical education (GME) training programs across the country are already entrusted with cultivating and assessing the knowledge, skills, and attitudes their trainees must attain upon completion of residency via the six domains of clinical competency as outlined by the Accreditation Council for Graduate Medical Education (ACGME). In the Next Accreditation System (NAS), measurement and reporting of these outcomes will be done via educational milestones (1). Many undergraduate medical education programs have already adopted the ACGME competencies. It is expected that milestones will also reach the undergraduate level and ‘contribute to a more seamless transition across the medical-education continuum’ (1). The initial milestones for entering residents will add performance-based vocabulary to conversations with medical schools about graduates’ preparedness for supervised practice (2).

On a GME level, assessing resident progress in achieving these domains has been and continues to be a challenge. The spectrum of conceptual models and tools utilized by individual programs speaks for the elusive nature of assessment, especially with regard to competencies outside of medical knowledge (3, 4).

The Dreyfus Framework has been one popular conceptual model on which many evaluation systems have been based (5). It describes phases of achieving professional expertise as novice, advanced beginner, competent, proficient, and expert. One negotiates each phase, moving from adhering to ‘context-free rules’ to developing ‘intuition’ in decision making and formulating plans. Some authors have called into question the application of the Dreyfus concept as an adequate way to understand complex implicit knowledge necessary in clinical thinking (6). Other developmental models have recently been developed, most notably the ‘Milestone model’ of resident assessment. These milestones were developed by an ACGME and American Board of Internal Medicine (ABIM) task force in 2007 (7).

Phrased in observable behavioral terms, the milestones operationalize the knowledge, skills, and attitudes a resident should demonstrate at different points in the 36 months of training across each competency (8). All training programs will eventually have to adopt the milestones in their evaluation tools to document trainee competence.

It is unclear what impact the transition to a Milestone-based model of assessment will have on the summative evaluations programs must report in the NAS. The aim of this study is to assess and report the distribution of resident performance ratings, across the 3 years of training for all core competencies, pre- and post-adoption of a Milestone model of assessment in one large internal medicine residency program.

We hypothesized that compared to a previous Dreyfus model of assessment, a Milestone model would add increased discriminative ability in assessing the six ACGME competencies for internal medicine residents in training.

## Methods

Our internal medicine training program is a large university-based program in New York with 146 trainees. House staff are evaluated at the end of every rotation by the attending they have worked with using an electronic global end of rotation form, among many other tools. The faculty in our Department of Medicine is diverse and includes a large component of subspecialty faculty, ambulatory faculty, and hospitalists.

In the academic year 2010–2011, our global rating form was based on a modified Dreyfus model of assessment and utilized a 5-point scale to evaluate trainees (1=beginner, 2=advanced beginner, 3=approaching competency, 4=competent, and 5=advanced competent). Full-time day hospitalists were trained on this evaluation method with a 2-h faculty development workshop in June of 2009. The session included a lecture presentation defining the model, a video tutorial, and a break out session where the faculty practiced using the Dreyfus model. Sporadic and repeated reinforcement of key concepts in the Dreyfus scale and its use in trainee evaluation occurred throughout the academic year during our monthly clinical competency meetings, which the hospitalists attended.

In the academic year 2011–2012, we shifted from the Dreyfus model of trainee assessment and began using a Milestone model. Thus, our global rating form changed to incorporate the ACGME milestones, again using a 5-point scale (i.e., 1=0–3 months, 2=6 months, 3=12 months, 4=18–24 months, 5=30–36 months). Representative behaviors expected of trainees for each of the time frames was taken from published milestones literature and provided on the form along with a hyperlink listing all milestones as described by the ACGME-ABIM task force. Full-time day hospitalists were trained on this evaluation method with a 2-h faculty development workshop in September of 2011. The session included a lecture presentation defining the Milestone model and a break out session where faculty were able to read and interpret the milestones in groups. Sporadic and repeated reinforcement of key concepts in the Milestone scale and its use in trainee evaluation occurred throughout the academic year during our monthly clinical competency meetings, which the hospitalists attended.

**Fig. 1:**
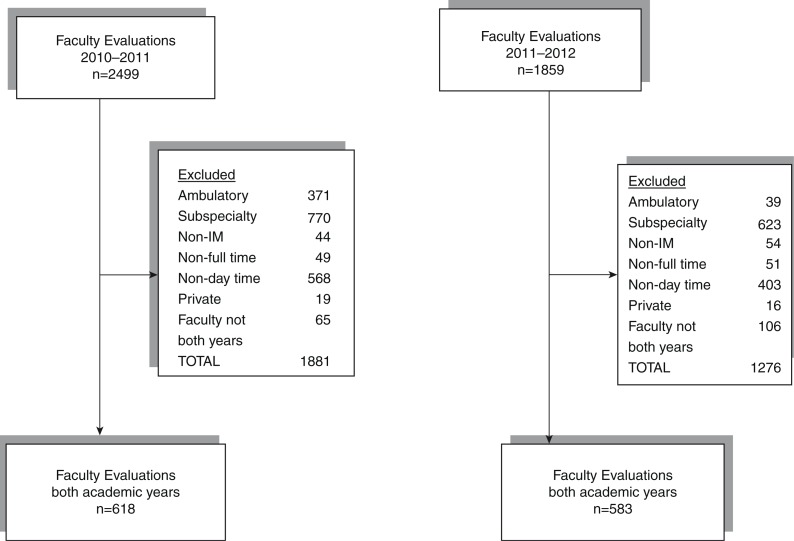
Diagram showing the inclusion and exclusion criteria of faculty evaluations.

In order to assess trainee progression along either developmental model (Dreyfus vs. Milestone), we analyzed all general medicine house staff evaluations completed by faculty from both academic years 2010–2011 and 2011–2012. Data pull of all faculty evaluations for both academic years included 4,358 evaluations. Exclusion criteria were applied as seen in Fig. 1. Ambulatory evaluations were excluded because the Milestone scale created for the ambulatory form was not 5 points and therefore not comparable to the prior Dreyfus model. Subspecialists, non-internal medicine faculty, non-full-time faculty, non-day time faculty, and faculty without evaluations in both academic years were excluded. When these exclusion criteria were applied, 1,201 evaluations were left for analysis (Fig. 1). The study was approved by the Hofstra North Shore-LIJ School of Medicine Institutional Review Board.

We analyzed all resident evaluations (*N*=1,201) that met our inclusion criteria. The primary outcomes were the distribution of scores on the 5-point evaluation form in the Dreyfus and Milestone model calculated for all six competencies. Means and standard deviations were computed for the primary outcomes. A repeated-measures MANOVA (Tables 1 and 2) was conducted using a mixed model approach for each of the scores within PGY year, between models (Dreyfus vs. Milestones) and evaluations (four 3-month periods per year) entered as fixed effects and evaluator and resident entered as random effects. Interactions between the three fixed effects were also entered. Since all of our hypotheses reflected differences contained within the three-way interaction, the interaction was not removed from the model even if it was non-significant. Based on a priori hypotheses to compare PGYs at each 3-month time period for each of the two models, to compare the models for each of the PGYs at each 3-month time period, and to compare each of the 3-month time periods within a model for each PGY year, significance levels were Bonferroni corrected such that *p*=0.001 for all pairwise comparisons. We plotted the 3-month time periods during the 2010–2011 and 2011–2012 academic years along the x-axis and average scores for each PGY class for each competency along the y-axis.

**Table 1:** Means (standard deviations) and significant pairwise comparisons from the ANOVA results for each competency score and total scores across the four evaluations (July–September, October–December, January–March, April–June) for PGY-1, PGY-2, and PGY-3 residents evaluated using the Dreyfus and Milestone models

Competency score	Evaluation	Model	PGY-1	PGY-2	PGY-3
Patient care	1 (*n*=102, 25, 36)	Dreyfus	2.60 (0.86)	3.88 (0.78)^a^	4.39 (0.64)^a^
	1 (*n*=123, 40, 37)	Milestone	1.76 (0.71)^b^	4.00 (0.55)^a^	4.92 (0.28)^a^,^c^
	2 (*n*=100, 31, 37)	Dreyfus	3.14 (0.88)^d^	4.16 (0.69)^a^	4.35 (0.75)^a^
	2 (*n*=95, 37, 20)	Milestone	2.41 (0.68)^b^,^d^	3.92 (0.43)^a^	4.95 (0.22)^a^,^c^
	3 (*n*=90, 22, 23)	Dreyfus	3.39 (0.91)^d^	4.27 (0.46)^a^	4.52 (0.51)^a^
	3 (*n*=83, 34, 15)	Milestone	2.82 (0.50)^b^,^d^,^e^	4.15 (0.44)^a^	4.80 (0.41)^a^
	4 (*n*=103, 21, 27)	Dreyfus	3.54 (0.81)^e^,^f^	4.14 (0.57)^a^	4.26 (0.59)^a^
	4 (*n*=59, 26, 14)	Milestone	3.24 (0.54),^d^,^e^,^f^	4.23 (0.43)^a^	5.00 (0.00)^a^
Medical knowledge	1 (*n*=102, 25, 36)	Dreyfus	2.60 (0.85)	3.84 (0.75)^a^	4.25 (0.60)^a^
	1 (*n*=123, 40, 37)	Milestone	1.84 (0.73)^b^	3.78 (0.62)^a^	4.76 (0.43)^a^,^c^
	2 (*n*=100, 31, 37)	Dreyfus	3.10 (0.83)^d^	4.10 (0.70)^a^	4.22 (0.75)^a^
	2 (*n*=95, 31, 20)	Milestone	2.45 (0.65)^b^,^d^	3.95 (0.33)^a^	4.90 (0.31)^a^,^b^,^c^
	3 (*n*=90, 22, 23)	Dreyfus	3.31 (0.86)	4.18 (0.50)^a^	4.43 (0.51)^a^
	3 (*n*=83, 34, 15)	Milestone	2.82 (0.52)^b^,^d^,^e^	4.09 (0.45)^a^	4.87 (0.35)^a^,^c^
	4 (*n*=104, 21, 27)	Dreyfus	3.50 (0.72)^e^,^f^	4.10 (0.70)^a^	4.22 (0.64)^a^
	4 (*n*=59, 26, 14)	Milestone	3.27 (0.49)^d^,^e^,^f^	4.15 (0.37)^a^	4.93 (0.27)^a^,^c^
Practice-based learning and improvement	1 (*n*=102, 25, 36)	Dreyfus	2.71 (0.86)	4.00 (0.58)^a^	4.31 (0.58)^a^
	1 (*n*=123, 40, 37)	Milestone	1.80 (0.65)^b^	3.90 (0.50)^a^	4.84 (0.37)^a^,^c^
	2 (*n*=98, 30, 37)	Dreyfus	3.19 (0.82)^d^	4.17 (0.53)^a^	4.24 (0.76)^a^,^b^
	2 (*n*=95, 31, 20)	Milestone	2.37 (0.65)^b^,^d^	3.92 (0.49)^a^	4.90 (0.31)^a^,^c^
	3 (*n*=90, 22, 22)	Dreyfus	3.41 (0.97)^d^	4.18 (0.50)^a^	4.55 (0.51)^a^
	3 (*n*=83, 34, 15)	Milestone	2.72 (0.53)^b^,^d^,^f^	4.09 (0.51)^a^	4.80 (0.41)^a^
	4 (*n*=104, 21, 27)	Dreyfus	3.54 (0.82)^d^,^e^	4.14 (0.73)^a^	4.15 (0.60)^a^,^b^
	4 (*n*=59, 26, 14)	Milestone	3.10 (0.36)^b^,^d^,^e^,^f^	4.19 (0.49)^a^	4.93 (0.27)^a^,^c^
Interpersonal and communication skills	1 (*n*=102, 25, 36)	Dreyfus	3.25 (1.03)	4.28 (0.61)^a^	4.53 (0.56)^a^
	1 (*n*=123, 40, 37)	Milestone	2.11 (0.82)^b^	4.20 (0.56)^a^	4.81 (0.40)^a^
	2 (*n*=100, 30, 37)	Dreyfus	3.78 (0.95)^d^	4.47 (0.57)^a^	4.38 (0.76)^a^
	2 (*n*=95, 37, 20)	Milestone	2.61 (0.79)^b^,^d^	3.95 (0.57)^a^	4.90 (0.31)^a^,^c^
	3 (*n*=89, 22, 22)	Dreyfus	3.85 (1.08)^d^	4.32 (0.48)	4.55 (0.51)^a^
	3 (*n*=83, 34, 15)	Milestone	3.01 (0.65)^b^,^d^,^e^	4.24 (0.50)^a^	4.87 (0.35)^a^
	4 (*n*=103, 21, 27)	Dreyfus	3.91 (0.86)^d^	4.43 (0.60)	4.26 (0.59)
	4 (*n*=59, 26, 14)	Milestone	3.49 (0.68)^b^,^d^,^e^,^f^	4.38 (0.50)^a^	4.93 (0.27)^a^
Systems-based practice	1 (*n*=102, 25, 36)	Dreyfus	2.73 (0.94)	3.92 (0.57)^a^	4.36 (0.54)^a^,^c^
	1 (*n*=123, 40, 37)	Milestone	1.93 (0.67)^b^	3.93 (0.53)^a^	4.65 (0.48)^a^,^c^
	2 (*n*=100, 31, 37)	Dreyfus	3.18 (0.90)^d^	4.16 (0.58)^a^	4.27 (0.80)^a^
	2 (*n*=95, 37, 20)	Milestone	2.47 (0.63)^b^,^d^	3.84 (0.44)^a^	4.80 (0.41)^a^,^c^
	3 (*n*=89, 21, 23)	Dreyfus	3.44 (1.03)^d^	4.29 (0.46)^a^	4.57 (0.51)^a^
	3 (*n*=83, 34, 15)	Milestone	2.75 (0.51)^b^,^d^	4.09 (0.51)^a^	4.73 (0.46)^a^
	4 (*n*=104, 21, 27)	Dreyfus	3.57 (0.76)^d^,^e^	4.19 (0.75)^a^	4.22 (0.64)^a^
	4 (*n*=59, 26, 14)	Milestone	3.15 (0.45)^b^,^d^,^e^,^f^	4.19 (0.40)^a^	4.93 (0.27)^a^,^c^
Professionalism	1 (*n*=102, 25, 36)	Dreyfus	3.19 (0.54)	3.60 (0.50)	3.69 (0.47)^a^
	1 (*n*=123, 40, 37)	Milestone	2.20 (0.90)^b^	4.18 (0.59)^a^,^b^	4.92 (0.28)^a^,^b^,^c^
	2 (*n*=98, 31, 37)	Dreyfus	3.39 (0.59)	3.52 (0.57)	3.54 (0.60)
	2 (*n*=95, 37, 20)	Milestone	2.75 (0.77)^b^,^d^	4.03 (0.60)^a^,^b^	5.00 (0.00)^a^,^b^,^c^
	3 (*n*=90, 22, 23)	Dreyfus	3.36 (0.68)	3.55 (0.51)	3.70 (0.47)
	3 (*n*=83, 34, 15)	Milestone	3.07 (0.66)^d^,^e^	4.26 (0.51)^a^,^b^	4.80 (0.41)^a^,^b^
	4 (*n*=104, 21, 27)	Dreyfus	3.49 (0.52)	3.76 (0.44)	3.41 (0.64)
	4 (*n*=59, 26, 14)	Milestone	3.49 (0.63),^d^,^f^	4.38 (0.50)^a^,^b^	4.93 (0.27)^a^,^b^
Overall	1 (*n*=102, 25, 36)	Dreyfus	2.64 (0.91)	3.88 (0.73)^a^	4.42 (0.60)^a^,^c^
	1 (*n*=123, 40, 37)	Milestone	1.88 (0.74)^b^	4.00 (0.51)^a^	4.86 (0.35)^a^,^c^
	2 (*n*=98, 30, 37)	Dreyfus	3.16 (0.84)^d^	4.19 (0.70)^a^	4.35 (0.75)^a^
	2 (*n*=95, 37, 20)	Milestone	2.52 (0.63)^b^,^d^	3.92 (0.43)^a^	4.95 (0.22)^a^,^c^
	3 (*n*=88, 20, 22)	Dreyfus	3.45 (0.96)^d^,^e^	4.25 (0.55)^a^	4.57 (0.51)^a^
	3 (*n*=83, 34, 15)	Milestone	2.86 (0.57)^b^,^d^	4.18 (0.46)^a^	4.80 (0.41)^a^
	4 (*n*=103, 21, 27)	Dreyfus	3.59 (0.77)^b^,^d^	4.14 (0.57)^a^	4.27 (0.60)^a^
	4 (*n*=59, 26, 14)	Milestone	3.19 (0.47)^b^,^d^,^e^,^f^	4.23 (0.43)^a^	5.00 (0.00)^a^,^c^

a Within model, significantly different from PGY-1

b within PGY year and month of evaluation, significantly different from Dreyfus

c within model, significantly different from PGY-2

d within PGY year and model, significantly (*p*<0.001) different from evaluation 1

e from evaluation 2

f from evaluation 3.

**Table 2:** MANOVA results for each of the competency and total scores

Scores	Model	PGY year	Evaluation	Model*PGY	Model*Evaluation	PGY*Evaluation	Model*PGY*Evaluation
Patient care	(1,948)=1.50, *p*=0.22	(2,948)=515.75, *p*<0.0001	(3,948)=22.31, *p*<0.0001	(2,948)=42.00, *p*<0.0001	(3,948)=1.88, *p*=0.13	(6,948)=18.03, *p*<0.0001	(6,948)=2.35, *p*=0.03
Medical knowledge	(1,949)=0.11, *p*=0.74	(2,949)=542.21, *p*<0.0001	(3,949)=28.35, *p*<0.0001	(2,949)=42.03, *p*<0.0001	(3,949)=2.17, *p*=0.09	(6,949)=13.49, *p*<0.0001	(6,949)=1.49, *p*=0.18
Practice-based learning and improvement	(1,945)=5.41, *p*=0.02	(2,945)=574.19, *p*<0.0001	(3,945)=19.76, *p*<0.0001	(2,945)=61.12, *p*<0.0001	(3,945)=3.19, *p*=0.02	(6,945)=13.97, *p*<0.0001	(6,945)=1.66, *p*=0.13
Interpersonal and communication skills	(1,945)=17.55, *p*<0.0001	(2,945)=290.32, *p*<0.0001	(3,945)=11.19, *p*<0.0001	(2,945)=51.75, *p*<0.0001	(3,945)=3.56, *p*=0.01	(6,945)=10.96, *p*<0.0001	(6,945)=1.81. *p*=0.09
System-based practice	(1,947)=9.11, *p*=0.0026	(2,947)=539.01, *p*<0.0001	(3,947)=22.30, *p*<0.0001	(2,947)=45.18. *p*<0.0001	(3,947)=2.95, *p*=0.03	(6,947)=10.30, *p*<0.0001	(6,947)=1.47, *p*=0.19
Professionalism	(1,947)=119.49, *p*<0.0001	(2,947)=246.54, *p*<0.0001	(3,947)=8.66, *p*<0.0001	(2,947)=135.35, *p*<0.0001	(3,947)=5.64, *p*=0.0008	(6,947)=11.42, *p*<0.0001	(6,947)=5.16, *p*<0.0001
Overall	(1,942) = 1.68, *p*=0.20	(2,942) = 501.56, *p*<0.0001	(3,942) = 21.13, *p*<0.0001	(2,942) = 40.37, *p*<0.0001	(3,942) = 1.84, *p*=0.14	(6,942) = 14.90, *p*<0.0001	(6,942) = 1.91, *p*=0.08

## Results

**Fig. 2:**
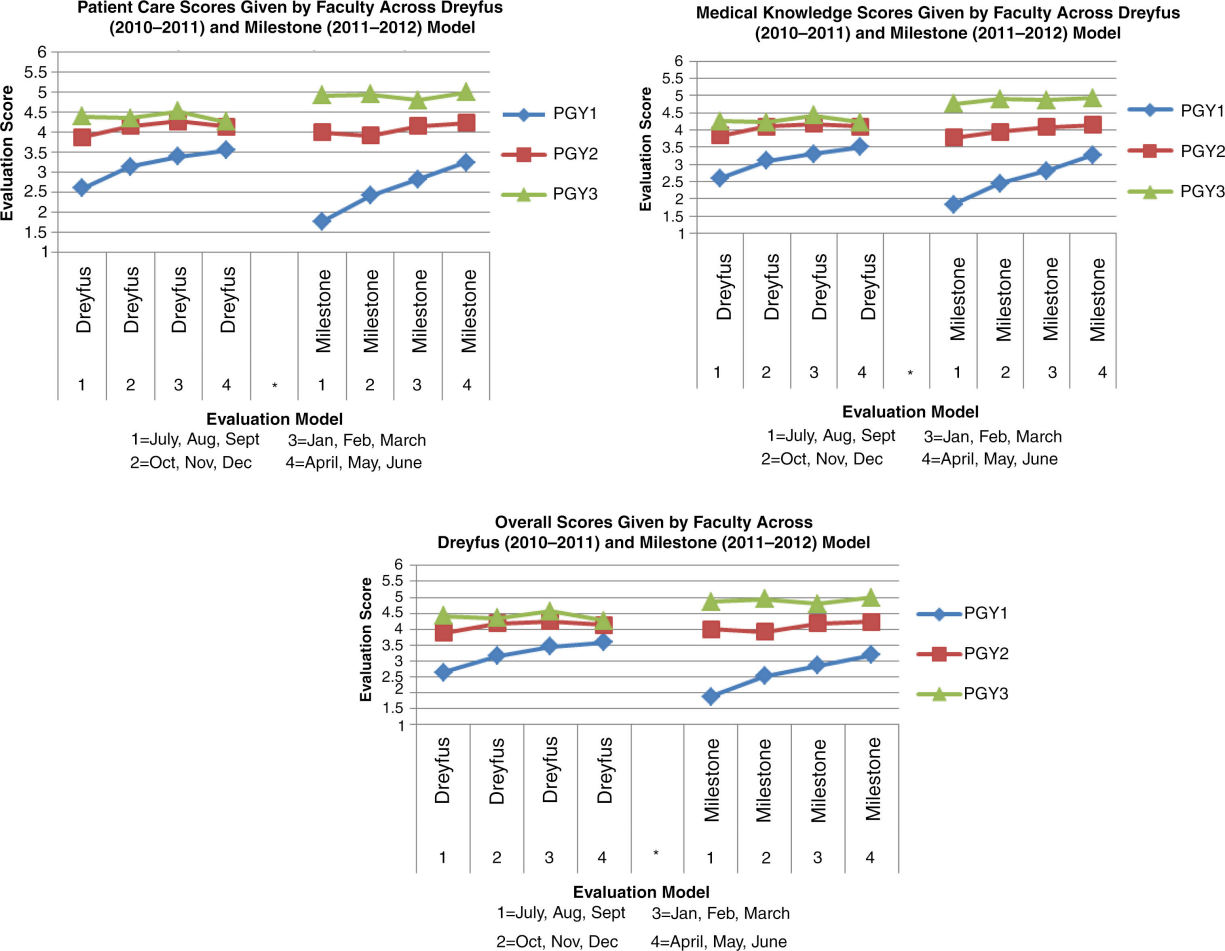
House staff evaluation scores done by full-time day faculty across the competencies patient care and medical knowledge and overall score across both evaluation models.

**Fig. 3:**
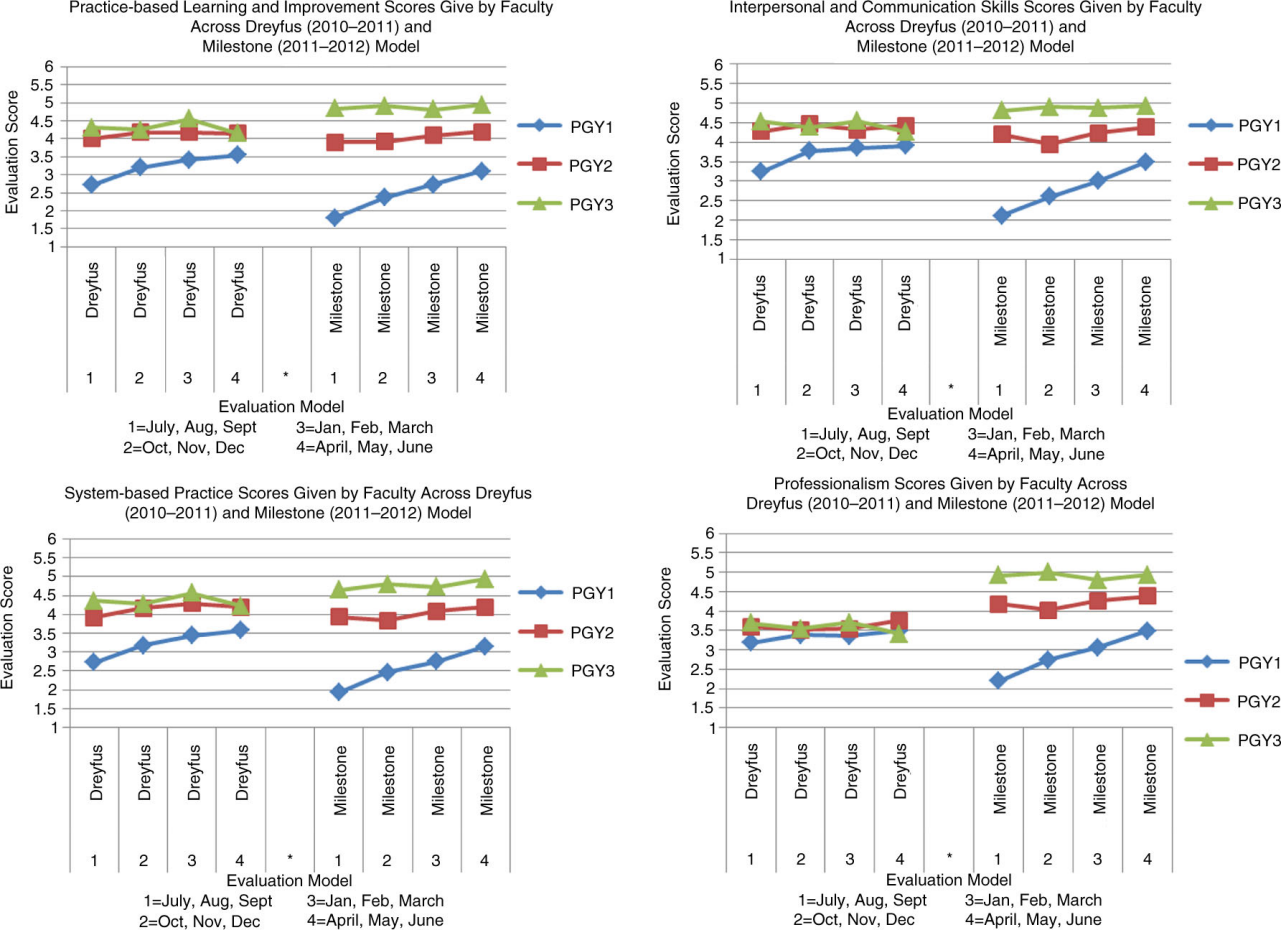
House staff evaluation scores done by full-time day faculty across the competencies practice-based learning and improvement, interpersonal and communication skills, systems-based practice and professionalism across both evaluation models.

Figures 2 and 3 display seven graphs depicting trainee skill acquisition (y-axis) over time (x-axis) in the Dreyfus and Milestone models as assessed by faculty meeting inclusion criteria. There is one graph for each competency. The average score for each PGY class at 3-month intervals across both evaluation models is plotted. These data are also depicted quantitatively in Table 1, with superscript letters indicating the following: within PGY year and model, significant differences from evaluation 1 (a), from evaluation 2 (b), and from evaluation 3 (c); within model, significant differences from PGY-1 (d) and from PGY-2 (e); and within PGY year and month of evaluation, significant difference from the Dreyfus model (f).

A larger separation of the scores between the PGY classes was seen in the Milestone model than in the Dreyfus model. There were no differences between PGY-2 and PGY-3 classes with the Dreyfus model although separation from PGY-1 was seen for all competencies except professionalism. In the Milestone model, we were able to achieve a much greater separation for all of the competencies, between all classes.

A steady progressive improvement in scores can be seen for the PGY-1 class in both models for all competencies; however, the Milestone model had a steeper curve. The PGY-2s and the PGY-3s displayed no progression in either model for all competencies. Thus, changing to the Milestone model did not change the apparent progression of skills acquisition in either the PGY-2 or PGY-3 class.

The Milestone model resulted in a wider use of the range in the 5-point scale compared to the Dreyfus model. Table 1 shows a range of mean scores from 2.6 to 5 and 1.76 to 5 in the Dreyfus and Milestone models, respectively.

The Milestone model resulted in a shift of the average scores for the PGY-1 and PGY-3 class. Figures 2 and 3 show that in the Milestone model compared with the Dreyfus model, the PGY-1 class had lower scores, the PGY-3 class scored higher and the PGY-2 class stayed the same.

## Discussion

All residency programs must report Milestone achievement directly to the ACGME in the NAS. When transitioning to a Milestone-based model, programs should assess if the resultant system is an improvement over prior models of documenting trainees’ developmental skills. One way to document this progression is to graph each resident's performance over time. One of the four possible conclusions can then be made: 1) the residents are progressing appropriately and the evaluation tool reflects that progression; 2) the residents are progressing appropriately, but the evaluation tool does not reflect the progression; 3) residents are not progressing appropriately and the evaluation tool captures the lack of progression; 4) residents are not progressing appropriately but the evaluation tool fails to capture the lack of progression. Programs can draw conclusions as to which of the four scenarios they feel represents their residents. If the evaluation systems are deemed to accurately represent skill acquisition, program directors can make decisions regarding remediation or advancement. If the evaluation systems are deemed invalid, they need to be changed. Analysis of our program's aggregated data indicated that in our program we were likely to be experiencing scenario number two.

Data analysis revealed four problems with how day time faculty utilized the global rating form in the Dreyfus model in our program. First, the scores for each class were not separate from each other and did not reflect the difference in clinical skills that faculty felt truly existed between classes. The PGY-2 and PGY-3 classes overlapped significantly in the Dreyfus model. In particular, all classes overlapped for professionalism. Assessing professionalism is hampered by varying definitions of the competency and thus is a concept that can be difficult to pin down and evaluate on single source tools (9). Shifting to the Milestone model, enhanced the discriminatory ability of the evaluator and allowed for a greater separation of the graphs as illustrated in Figs. 2 and 3. In our opinion, this scenario better mirrors the difference in clinical skills among our PGY classes and is most obvious in the competency of professionalism.

The second problem with the Dreyfus model was an upward progression in slope for the PGY-1 class, but virtually no slope in the PGY-2 and PGY-3 classes. The Milestone model created a steeper slope for the PGY-1 class. However, the PGY-2 and PGY-3 classes remained flat, albeit to a lesser degree (Figs. 2 and 3, Table 1).

Third, analysis of the scores in the Dreyfus model showed that faculty was not using the full range of the 5-point scale. This is a well-recognized limitation of global rating forms (10). With the Milestone evaluation form, we were pleased to see a greater use of the full range of the scale. In particular, evaluators more frequently rated interns with a score of 1 in the Milestone model (0–3 months) than in the Dreyfus model (i.e., beginner).

Finally, the average score in July for the PGY-1 class was significantly lower in the Milestone model compared with the Dreyfus model, as seen in Table 1. This result reflects the faculty's increased willingness to use the lower end of the rating scale thereby counteracting the well-known ceiling effect, where ratings are primarily clustered in the highest category of the scale, as seen with many global rating forms (11).

It is worthwhile to note that the results we observed, regardless of model used, are a reflection of the faculty's ability to observe and document resident behavior. It is well recognized that observation of trainees by faculty is at best suboptimal (12). Milestones are meant to help focus faculty's observation skills.

To our knowledge, this is the first study to report on how aggregate data may change as programs transition to Milestone reporting in the NAS. By keeping a 5-point scale for both academic years, we were able to directly compare scores and graphs. We have provided two full academic years’ worth of aggregate data using a large number of faculty evaluations. By critically analyzing our data, we were able to see the improvements milestones have made in distinguishing the PGY-2 and PGY-3 classes from each other over the Dreyfus model but also where improvement still needed to be made. We did not expect a lack of progression to be seen in these classes. This discovery will guide us to make changes once again. We were especially pleased to see that the Milestone model was able to separate scores even for the competency of professionalism, traditionally a difficult competency to evaluate on a rating form.

We acknowledge certain limitations of our study. It was conducted at one site with milestones only incorporated into our global end of rotation rating form. Our program has multiple other evaluation tools that were not included in this analysis. Global rating forms have limited intrarater and interrater reliability and lack reproducibility (13, 14). They can be subject to central tendency errors, where the evaluators fail to use the entire rating scale, and the ‘halo’ effect, where evaluators’ biases about an initial impression by one component of a resident is extended to other aspects of the resident's performance (8, 13). The last limitation of this study was our need for more robust faculty development. Faculty development was present in the pilot phases of both our models, but sparse throughout the academic years. Faculty development is pivotal in operationalizing whatever evaluation system one chooses and has been shown to be most effective when done in a comprehensive and continuous fashion (13, 14).

Despite its limitation, the global end of rotation rating form, with its low cost, flexibility and ease of use, will likely be a popular evaluation tool for future use (8, 10). Using the tool successfully requires studying the distribution of scores achieved and reflecting on whether these scores truly reflect trainee competence. Our program aimed to achieve a greater distribution of scores using a 5-point scale with milestones as anchors. With the Milestone anchors there was a greater spread but even with this widened distribution of scores there was still room to improve. The ACGME, with its move toward reportable milestones for each trainee on a 9-point scale will undoubtedly also assess trainee progression over time graphically. We are pleased to have 2 years of experience doing the same. Our program has subsequently changed to an 8-point scale to give faculty more of a chance to differentiate behaviors between the PGY-2 and PGY-3 classes. Further change will continue as we adjust to the ACGME reportable milestones in the NAS. It will be important for the ACGME to similarly graph its nationwide aggregate trainee performance data overtime. Programs will need to continuously self reflect upon their evaluation systems and adjust them until a ‘true’ accounting of trainees skill set is represented.

Graduate training programs are just beginning to learn how to incorporate milestones into their evaluation tools. Full incorporation will take time and will rely heavily on faculty buy-in and faculty development. Only through a thoughtful reflection of trainee evaluations and rating scales will programs be able to successfully document competency in the future generation of physicians.
